# *Rhabdophis tigrinus* is not a pit viper but its bites result in venom-induced consumptive coagulopathy similar to many viper bites

**DOI:** 10.1186/s40560-014-0043-6

**Published:** 2014-07-31

**Authors:** Anjana Silva, Toru Hifumi, Atsushi Sakai, Akihiko Yamamoto, Masahiro Murakawa, Manabu Ato, Keigo Shibayama, Akihiko Ginnaga, Hiroshi Kato, Yuichi Koido, Junichi Inoue, Yuko Abe, Kenya Kawakita, Masanobu Hagiike, Yasuhiro Kuroda

**Affiliations:** Department of Parasitology, Faculty of Medicine and Allied Sciences, Rajarata University of Sri Lanka, Anuradhapura, Saliyapura 50008 Sri Lanka; Emergency Medical Center, Kagawa University Hospital, 1750-1 Ikenobe, Miki, Kita, Kagawa 761-0793 Japan; The Japan Snake Institute, Yabutsuka 3318, Ota, Gumma 379-2301 Japan; Department of Bacteriology II, National Institute of Infectious Disease, Gakuen 4-7-1, Musashimurayama-shi, Tokyo 208-0011 Japan; Department of Internal Medicine, Kaizuka Hospital, Hakosaki 7-7-27, Higashi-ku, Fukuoka 812-0053 Japan; Department of Immunology, National Institute of Infectious Disease, Toyama 1-23-1, Shinjuku-ku, Tokyo 162-8640 Japan; The Chemo-Sero-Therapeutic Research Institute (KAKETSUKEN), 1-6-1 Okubo, Kita-ku, Kumamoto-shi, Kumamoto 860-8568 Japan; Division of Critical Care Medicine and Trauma, National Hospital Organization Disaster Medical Center, 3256 Midori-cho, Tachikawa, Tokyo 190-0014 Japan; Division of Critical Care Medicine and Trauma, Yamanashi Prefectural Central Hospital, 1-1-1 Fujimicho, Kofu, Yamanashi 400-8506 Japan

**Keywords:** Viperidae, Colubridae, Natricinae, *Rhabdophis*, Coagulopathy

## Abstract

As a response to the recent article by Hifumi et al. published in the *Journal of Intensive Care*, the present correspondence clarifies the family-level taxonomy of the yamakagashi (*Rhabdophis tigrinus*). Further, the relevance of the term ‘venom-induced consumptive coagulopathy,’ instead of disseminated intravascular coagulation, in describing the procoagulant coagulopathy of *R. tigrinus* is highlighted.

## Correspondence

Anjana Silva

Dear Editor,

I read with interest, the research article titled ‘Clinical characteristics of yamakagashi (*Rhabdophis tigrinus*) bites: a national survey in Japan, 2000–2013’ by Hifumi et al. [1] published recently in the *Journal of Intensive Care*. Underreporting of snakebites has been a challenge in confronting the global burden of snakebite. Therefore, studies such as Hifumi et al. are with high value for the field of clinical toxinology as the authors have comprehensively described nine cases of *R. tigrinus* bites by providing clues for the pathophysiology of envenoming.

One major issue in the clinical reporting of snakebites is lack of emphasis on the taxonomic identity of the offending snakes, often leading to confusions [2]. Since the clinical reporting of snakebites is primarily done by the clinicians, this could be expected. In Hifumi et al. [1], *R. tigrinus* has been erroneously mentioned as a pit viper species (family: Viperidae, subfamily: Crotalinae). *R. tigrinus* has long been placed within the family Colubridae, under the subfamily Natricinae and genus *Rhabdophis* [3,4], and therefore is not a pit viper but is a colubrid. Although some authors have suggested treating natricines as a separate family called Natricidae [5], recent molecular work confirmed the placement of natricines within the Colubridae as a subfamily [6]. All vipers (family: Viperidae) are venomous and possess front fangs. Pit vipers are an evolutionarily distinct group within the family Viperidae. As opposed to viperids, most of the snakes of the largest snake family Colubridae are non-venomous and do not possess true venom glands or venom delivery systems. Few mildly venomous snake groups within this family, such as the genus *Rhabdophis*, possess rear fangs as well as Duvernoy's glands which produce venom-like secretions and associated low-pressure venom delivery systems [7].

The pro-coagulant nature of *R. tigrinus* venom is primarily due to its prothrombin activating effects and weak thrombin-like effects. This leads to hypofibrinogenemia resulting hemorrhage in envenomed patients [1,8]. However, Hifumi et al. [1] described the above pathological process as the disseminated intravascular coagulation (DIC) with fibrinolytic phenotype. Interestingly, this pathological process has been described with the term ‘venom-induced consumptive coagulopathy’ (VICC) seen in many viperid snake groups (including many pit vipers) and Australian elapids. Although VICC is similar to DIC in many ways, VICC differs from the former by not usually resulting in end-organ effects, rapid onset and resolution, and the difference in the mechanism of initiation [9]. Hence, I suggest that the term VICC [9] is more suitable than DIC used by the authors to describe the coagulopathy due to *R. tigrinus* bites.

## Response

Toru Hifumi, Atsushi Sakai, Akihiko Yamamoto, Masahiro Murakawa, Manabu Ato, Keigo Shibayama, Akihiko Ginnaga, Hiroshi Kato, Yuichi Koido, Junichi Inoue, Yuko Abe, Kenya Kawakita, Masanobu Hagiike, Yasuhiro Kuroda

Dear Editor,

We thank Dr. Silva for his comments on our article.

We completely agree that *Rhabdophis tigrinus* is a rear-fanged venomous snake present in paddy fields [10] and that its venom shows strong plasma coagulant activity, with prothrombin activating effects and weak thrombin-like effects that result in hemorrhagic symptoms [8]. Because *R. tigrinus* has no grooved fangs, envenomation does not occur in most bites; therefore, *R. tigrinus* has long been considered nonvenomous [10,11].

However, we totally disagree with his suggestion that ‘venom-induced consumptive coagulopathy’ (VICC) is a more suitable term than ‘disseminated intravascular coagulation’ (DIC) with a fibrinolytic phenotype used to describe coagulopathy due to *R. tigrinus* bites.

First, we would like to provide the details of coagulation markers of case 5 in our previous studies [1,11]. A healthy 40-year-old man was admitted with severe coagulopathy that developed after *R. tigrinus* bites. On admission, he demonstrated significantly elevated levels of thrombin-antithrombin III complex (TAT, 60 ng/mL; normal range, <3–4 ng/mL), plasmin-alpha 2-plasmin inhibitor complex (PIC, 22.3 μg/mL; normal range, <0.8 μg/mL), and fibrinogen degradation products (FDPs, 592 μg/mL). He subsequently developed severe hypofibrinogenemia (50 mg/dL). Antivenom was administered 28 h after being bitten; after which, his hemorrhagic symptoms resolved. By day 3 of admission, scabs had formed over the bite wounds. Furthermore, his fibrinogen levels increased to >100 mg/dL, while his TAT, PIC, and FDP levels normalized. He was discharged on day 6 of admission. These coagulation markers fulfilled the diagnostic criteria for DIC with a fibrinolytic phenotype [12].

Second, Dr. Silva cited a research conducted by Isbister who suggested that a snakebite does not cause DIC but instead causes coagulopathy and thrombotic microangiopathy in snake envenoming; this was reportedly because VICC was not characterized by important features of DIC, such as evidence of systemic microthrombi and end-organ failure [9].

A similar discussion has been made in the context of trauma, which also induces DIC with a fibrinolytic phenotype in the acute phase. Rizoli et al. conducted a prospective study and concluded that within 24 h of trauma, most severely injured patients have DIC scores ‘suggestive of’ or of ‘overt DIC’ in the International Society on Thrombosis and Haemostasis (ISTH) score but have no anatomopathologic evidence of DIC. Considering that pathologic findings are the gold standard for diagnosis, then, DIC is exceptionally uncommon, and the ISTH score should not be used for trauma [13].

Asakura described important principals that enhanced fibrinolytic-type DIC (DIC with a fibrinolytic phenotype) is associated with marked activation of fibrinolysis corresponding to activation of coagulation. Fibrinolysis is strongly activated, hemostatic plugs (thrombi due to hemostasis) are more easily dissolved, and bleeding symptoms tend to be severe. However, organ dysfunction seldom occurs [12].

Therefore, it is obvious that we cannot find evidence of systemic microthrombi and that patients do not develop organ dysfunction in the phase of DIC with a fibrinolytic phenotype.

Third, the previous literature was limited by the sample size (nine cases) [1]; therefore, we are now preparing the next manuscript. We further analyzed 25 cases from the previous study [1]. Over the 43-year study period (1970–2013), 34 patients were identified, and the number of patients developing renal failure who required hemodialysis was significantly lower in the antivenom group than in the without-antivenom group (5.3% vs 40.0%, *P* = 0.03). We assume that DIC with fibrinolysis phenotype does not persist throughout hospitalization and may be limited to the acute phase of injury for *R. tigrinus* bites. Renal failure requiring hemodialysis developed at a later phase of injury in 40% of patients not receiving antivenom. Examination of renal pathology revealed that glomerular fibrin thrombi and tubular necrosis were responsible for renal failure associated with *R. tigrinus* bites [14]. Indeed, Gando et al. reported that 24–48 h after severe traumatic injury, DIC with a fibrinolytic phenotype developed into that with a thrombotic phenotype, therefore resulting in fatal multiple organ dysfunction syndromes [15,16].

In conclusion, we continue to assert that *R. tigrinus* bites induced DIC with a fibrinolytic phenotype.
